# Discriminant WSRC for Large-Scale Plant Species Recognition

**DOI:** 10.1155/2017/9581292

**Published:** 2017-12-25

**Authors:** Shanwen Zhang, Chuanlei Zhang, Yihai Zhu, Zhuhong You

**Affiliations:** ^1^Department of Information Engineering, Xijing University, Xi'an 710123, China; ^2^Tableau Software, Seattle, WA 98103, USA

## Abstract

In sparse representation based classification (SRC) and weighted SRC (WSRC), it is time-consuming to solve the global sparse representation problem. A discriminant WSRC (DWSRC) is proposed for large-scale plant species recognition, including two stages. Firstly, several subdictionaries are constructed by dividing the dataset into several similar classes, and a subdictionary is chosen by the maximum similarity between the test sample and the typical sample of each similar class. Secondly, the weighted sparse representation of the test image is calculated with respect to the chosen subdictionary, and then the leaf category is assigned through the minimum reconstruction error. Different from the traditional SRC and its improved approaches, we sparsely represent the test sample on a subdictionary whose base elements are the training samples of the selected similar class, instead of using the generic overcomplete dictionary on the entire training samples. Thus, the complexity to solving the sparse representation problem is reduced. Moreover, DWSRC is adapted to newly added leaf species without rebuilding the dictionary. Experimental results on the ICL plant leaf database show that the method has low computational complexity and high recognition rate and can be clearly interpreted.

## 1. Introduction

Plant species automatic identification via computer vision and image processing techniques is very useful and important for biodiversity conservation. The plant species can be recognized by their flowers, leaves, fruits, barks, roots, and stems [1–3]. Leaf is often used for plant species identification. A leaf image can be characterized by its color, texture, and shape. In particular, leaf shape contains a lot of classifying features, such as leaf tip, blade, leaf base, teeth, veins, apex, margin, leafstalk, insertion point, and contour. The availability and universality of relevant technologies, such as digital cameras, smart phone, Internet of things (IOT), remote access to databases, and many effective algorithms and techniques in image processing and machine learning make leaf based automatic plant species identification become reality. Mata-Montero and Carranza-Rojas [4] summed up the recent efforts in the plant species recognition using computer vision and machine learning techniques, and the main technical issues for efficiently identifying biodiversity. Wäldchen and Mäder [5] introduced 120 peer-reviewed studies in the last 10 years from 2005 to 2015 and compared the existing methods of plant species identification based on the classification accuracy achieved on the public available datasets. Munisami et al. [6] proposed a plant recognition method by extracting a lot of features such as the leaf length, width, area, perimeter, hull perimeter, hull area, color histogram, and centroid-based radial distance. Chaki et al. [7] proposed a method of characterizing and recognizing plant leaves using a combination of texture and shape features. The extracted features are invariant to translation, rotation, and scaling factors. Different from the existing studies which target at simple leaves, Zhao et al. [8] proposed a plant identification method to accurately recognize both simple and compound leaves. de Souza et al. [9] presented a plant identification method by combining shape descriptor, feature selector, and classification model. The extracted shape descriptor is invariant to rotation, translation, scale, and start point of the edge centroid distance sequences. Liu and Kan [10] extracted a lot of texture and shape features for leaf classification, including Gabor filters, local binary patterns, Hu moment invariants, gray level cooccurrence matrices, and Fourier descriptors and applied deep belief networks with dropout to classify plant species. Vijayalakshmi and Mohan [11] extracted gray level cooccurrence matrix (GLCM) and local binary pattern (LBP) features for leaf classification. Pradeep Kumar et al. [12] presented a leaf recognition system using orthogonal moments as shape descriptors and histogram of oriented gradients (HOG) and Gabor features as texture descriptors.

**Figure 1:**
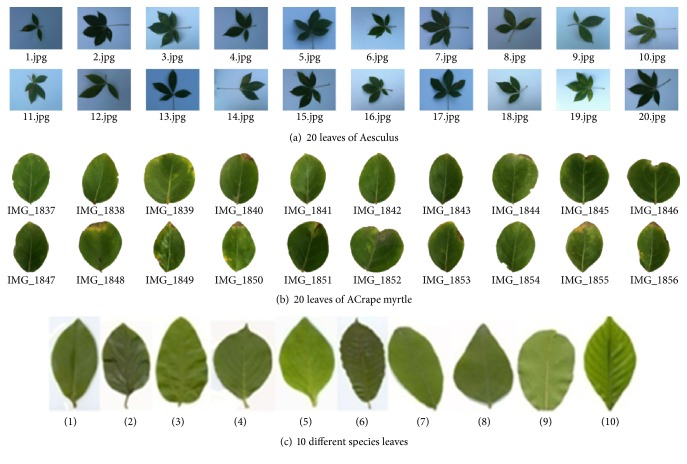
Plant leaf examples.

Although many leaf based plant species recognition methods have been proposed, this research still remains challenging. The reasons are that (1) the plant leaves are very various, complex, and changeable with seasons, and a leaf image can be taken under arbitrary position and the relative poses of its petiole and blade are often various in the different leaf images; (2) in nature, the leaf shapes have large intraclass difference (as shown in Figures 1(a) and 1(b)) and high interclass similarity (as shown in Figure 1(c)).

Recently, sparse representation based classification (SRC) has received significant attention and has been proven to be advantageous for various applications in signal processing, computer vision, and pattern recognition [13, 14]. SRC is robust to occlusion, illumination, and noise and has been applied to plant species identification and achieved better recognition results. Hsiao et al. [15] proposed two SRC based leaf image recognition frameworks for plant species identification and experimentally compared them. In the frameworks, an overcomplete dictionary is learned for sparsely representing the training images of each species. Jin et al. [16] proposed a plant species identification method using sparse representation (SR) of leaf tooth features. In the method, four leaf tooth features are extracted and plant species is recognized by SRC. In practice, however, the existing SRC and WSRC would fail in leaf based large-scale plant species recognition, because it is time-consuming to solve the *l*_1_-norm minimization problem in a dictionary composed of all training data across all classes. Several modified local SRC algorithms have been proposed to reduce the computational cost of SRC [17–19]. In these methods, the SR problem is performed for each test sample in its local neighborhood set. However, the constructed dictionary is exceptionally dependent on the test sample. Elhamifar and Vidal [20] proposed a structured SR for face recognition. They tried to look for the SR of a test sample using the minimum number of groups from the dictionary. The task of WSRC is to measure the significance of each training sample in representing the test samples. It can preserve the locality and similarity between the test sample and its neighbor training samples while looking for the sparse linear representation [21]. In classical SRC, WSRC, and their modified approaches, to achieve good sparse performance, all training samples are employed to build the overcomplete dictionary [13, 14]. However, it is time-consuming to solve the SR problem through the overcomplete dictionary, especially in large-scale image recognition task. To improve the performance of SRC, a lot of small-size dictionary schemes have been developed. Wright et al. [22] manually selected a few training samples to construct the dictionary. Mairal et al. [23] learned a separate dictionary for each class. Zhang et al. [24] presented a dictionary learning model to improve the SR performance. The test sample is recognized by only the reconstruction error of each class-specific subdictionary, instead of the features from all classes in the common subdictionary. Sun et al. [25] presented a dictionary learning model for image classification by learning a class-specific subdictionary for each class, where the subdictionary is shared by all classes.

Inspired by the recent progress of plant species identification and WSRC, we propose a discriminant weighted SRC (DWSRC) for large-scale plant species identification. In DWSRC, we calculate the sparse representation of the test example that involves the minimum number of training samples, instead of employing all the training data.

The main contribution of this paper is listed as follows:

(1) In DWSRC, instead of constructing the *l*_1_-norm minimization problem by employing all of the training samples, we solve a similar problem that involves the minimum number of training samples. Thus the computational cost to deal with the large-scale plant species recognition task will be significantly reduced.

(2) The side-effect of the noisy data and outliers on the classification decision can be eliminated through selecting the similar class to sparsely represent the test sample.

(3) DWSRC integrates the sparsity, and both local and global structure information of the training data into a unified framework, and considers more attention to the training data that are similar to the test sample in representing the test sample.

(4) DWSRC integrates data structure information and discriminative information.

The rest of this paper is organized as follows. In Section 2, we briefly review SRC, weighted SRC (WSRC), and overcomplete dictionary.  Section 3 presents a discriminant weighted SRC (DWSRC) approach for large-scale plant species identification.  Section 4 reports the experiment results on the popular leaf image database. Finally, Section 5 concludes this paper.

## 2. Related Work

### 2.1. SRC and WSRC

The goal of SRC and WSRC is to sparsely represent the test sample by the smallest number of nonzero coefficients in terms of the overcomplete dictionary and assign the test sample to the class with the minimum reconstitution error. Suppose there are *n* training samples *X* = [*x*_1_, *x*_2_,…, *x*_*n*_] ∈ *R*^*n*×*m*^ belonging to *C* classes and *y* ∈ *R*^*m*^ is a test sample. The task of SRC is to solve the following *l*_1_-norm minimization problem:

(1) a′=arg mina y−Aa22+μa1,

where *A* is an overcomplete dictionary built by all of the training samples, *a* = [*a*_1_, *a*_2_,…, *a*_*n*_]^*T*^ is a SR coefficient vector, *a*_*i*_  (*i* = 1,2,…, *n*) is the *i*th SR coefficient of *x*_*i*_, and *μ* > 0 is a scalar regularization parameter.

The weights of the training samples are often calculated by the distance or similarity between the test sample and each training sample. Similar to SRC, WSRC solves a weighted *l*_1_-norm minimization problem:

(2) a^=arg mina y−Aa22+μWa1,

where *W* is a block-diagonal weighted matrix.

The minimal problems of (1) and (2) can be solved by the sparse subspace clustering algorithm [26]. Ideally, the obtained SR coefficients are sparse, and thus the test sample can easily be assigned to that class [27]. Then *y* is assigned to the class with the minimum reconstruction error among all of the classes, which is defined as follows:

(3) identityy=argminc=1,2,…,C y−Aca^c2,

where ${\hat{a}}^{c}$ is the coefficient subvector corresponding to the* c*th training class in $\hat{a}$.

### 2.2. Dictionary of SRC

SRC and WSRC aim to find a sparse representation of the input data in the form of a linear combination of basic elements. These elements are called atoms and they compose a dictionary. Atoms in the dictionary are not required to be orthogonal, and they might be an overcomplete spanning set. Although SRC, WSRC, and their improved methods have been widely used in various practical applications, how to construct the dictionary is still a challenging issue in image processing. Many schemes have been proposed to efficiently learn the overcomplete dictionary by enforcing some discriminant criteria and achieve impressive performances on image classification, but most of the existing modified SRC methods are complex and difficult to be implemented [23, 24]. Moreover, most of these methods assume that the training samples of a particular class approximately form a linear basis for any test sample belonging to that class [18, 22–25]. But in nature, there are a lot of interclass similar leaves, such as in Figure 1(c). In particular, it is time-consuming to implement these methods on the large-scale database.

## 3. Discriminant WSRC for Large-Scale Plant Species Recognition

Based on the global and local SR, several WSRC and group SRC algorithms are proposed to suppress the small nonzero coefficients. Similar to SRC, these approaches assume that the samples from a single class lie on a linear subspace, but we do not know the class label of a test sample. Anyway, it is important to choose the “optimal” training samples to sparsely represent the test sample. To improve the performance of SRC, a discriminant WSRC (DWSRC) method is proposed for large-scale plant species recognition, given *n* training leaf images {*x*_1_, *x*_2_,…, *x*_*n*_} and *m* representative leaf images with large different shapes such as Linear, Oblong, Elliptic, and Ovate, where *t*_*j*_  (*j* = 1,2,…, *m*) is the* j*th representative image. WSRC is introduced in this section.

### 3.1. Subdictionary Construction

**Figure 2:**
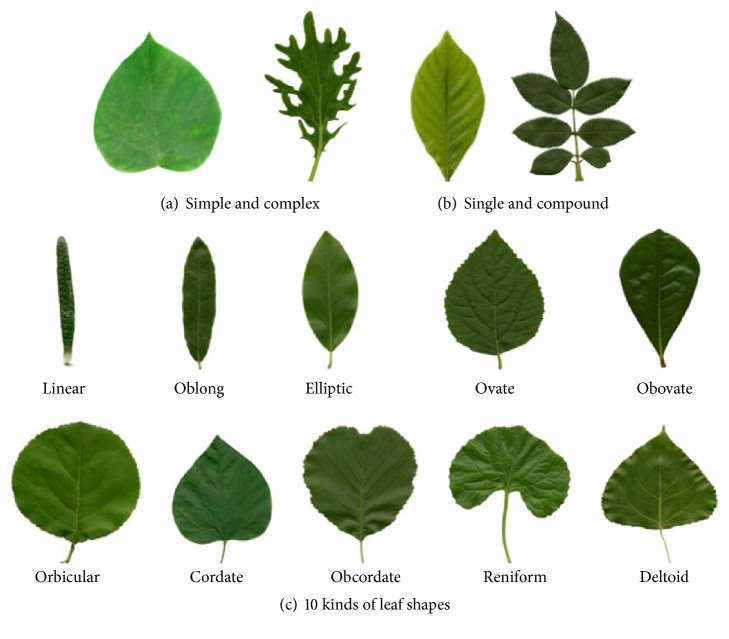
Plant leaf examples.

In nature, there are more than 400,000 kinds of plant species. Although there are various leaves in size, shape, color, and texture, we can divide all leaves into several similar classes by different leaf shape representation criteria, such as (1) Simple and Complex; (2) Single and Compound; (3) Linear, Oblong, Elliptic, Ovate, Obovate, Orbicular, Cordate, Obcordate, Reniform, Deltoid, and others, as shown in Figure 2.

The Gaussian kernel distance can capture the nonlinear information within the dataset, to measure the distance or similarity between the samples. Suppose there are two leaf images *x*, *y*, and their similarity is defined by the Gaussian kernel distance:

(4) $$\begin{array}{c} s\left(\;x,y\;\right)=\exp\left(\;-\frac{{\left\|x-y\right\|}^{2}}{2\beta^{2}}\;\right), \end{array}$$

where *β* is the Gaussian kernel width to control the excessive distance between *x* and *y* and ‖*x* − *y*‖ is the Euclidean distance between *x*, *y*.

In this section, any original leaf image is transformed into gray image or binary image. In fact, any gray or binary image is a 2D matrix. Thus ‖*x* − *y*‖ is the Euclidean distance between two matrices *x*, *y*.

Given *n* training leaf images {*x*_1_, *x*_2_,…, *x*_*n*_}, in order to divide these images into *m* similar classes, we find *m* representative leaf images with large different shapes such as Linear, Oblong, Elliptic, and Ovate, where *t*_*j*_  (*j* = 1,2,…, *m*) is the* j*th representative image. Then, we set up a threshold *T*. If *s*(*x*_*i*_, *t*_*j*_) ≥ *T*, *x*_*i*_ is assigned to the* j*th similar class; if *s*(*x*_*i*_, *t*_*j*_) < *T* for any *t*_*j*_  (*j* = 1,2,…, *m*), *x*_*i*_ does not belong to *m* given similar classes. It is assigned to the (*m* + 1)th similar class. Then *n* leaf images are roughly divided into *m* + 1 similar classes.

We concatenate each leaf image of *m* similar class as a one-dimensionality vector and construct *m* + 1 subdictionaries for the following WSRC algorithm by all these vectors; namely, *A*_*j*_ ∈ *R*^*n*_*j*_×*n*^  (*j* = 1,2,…, *m* + 1), where *n* is the dimensionality of the leaf image vector and *n*_*j*_ is the leaf number of the* j*th similar class, and each column of *A*_*j*_ is a vector corresponding to a leaf image.

### 3.2. Discriminant WSRC

SRC can ensure sparsity, while it loses the local information of the training data; WSRC can preserve the sparsity and the similarity between the test sample and its neighbor training data, while it ignores the prior global structure information of the training data. We proposed a discriminant WSRC (DWSRC) scheme to improve the classifying performance of WSRC by integrating the sparsity, and both locality and global structure information of the training data into a unified framework.

Given a test leaf image *y* and a threshold *T*_1_, by (4), we calculate the similarity *s*(*y*, *t*_*j*_)  (*j* = 1,2,…, *m*) between *y* and the* j*th representative leaf image *t*_*j*_  (*j* = 1,2,…, *m*). If *s*(*y*, *t*_*r*_) > *T*_1_, choose the *r*th similar class as the candidate similar class with the maximum similarity for classifying *y*, and select the corresponding *r*th subdictionary *A*_*r*_ as the candidate subdictionary. Similar to the general WSRC, DWSRC solves the following weighted *l*_1_-norm minimization problem,

(5) a′=arg mina y−Ara22+μWra1,

where *W*_*r*_ ∈ *R*^*n*_*r*_×*n*_*r*_^ is a weighted diagonal matrix, *n*_*r*_ is the leaf image number of the *r*th similar class, and the diagonal elements *w*_*r*1_, *w*_*r*2_,…, *w*_*rn*_*r*__ of *W*_*r*_ are the Gaussian kernel distances between *y* and the candidate samples, which are defined as follows:

(6) $$\begin{array}{c} w_{ri}=\exp\left(\;\frac{{\left\|y-x_{ri}\right\|}^{2}}{2\beta_{1}^{2}}\;\right), \end{array}$$

where *x*_*ri*_ is the *i*th vector of the candidate similar class and *β*_1_ is the Gaussian kernel width to adjust the weight decay speed.

In (5), *W*_*r*_ can penalize the distance between *y* and each training sample of the candidate similar class. Its objective is to avoid selecting the training samples that are far from the test sample to represent the test sample. Because the sample numbers of the different similar class are different, after obtaining the SR coefficient vector *a*, we compute the average reconstruction error of each class in the candidate similar class as follows:

(7) $$\begin{array}{c} m_{c}\left(\;y\;\right)=\frac{1}{n_{c}}{\left\|\;y-A_{r}a^{c}\;\right\|}_{2}, \end{array}$$

where *a*^*c*^ is the coefficient subvector associated with the* c*th training class in *a* and *n*_*c*_ is the vector number of the* c*th class in the candidate similar class.

Then the test image is labeled to the class that owns the minimum normalized reconstruction error *m*_*c*_(*y*).

In classical WSRC [21], if the diagonal elements *w*_1_, *w*_2_,…, *w*_*n*_ of weighted diagonal matrix *W* is defined as follows,

(8) $$\begin{array}{c} w_{i}=\left\{\begin{array}{cc} s\left(x_{i},y\right), & \text{if }s\left(x_{i},y\right)>T\\ 0, & otherwise, \end{array}\right. \end{array}$$

then WSRC is DWSRC.

**Figure 3:**
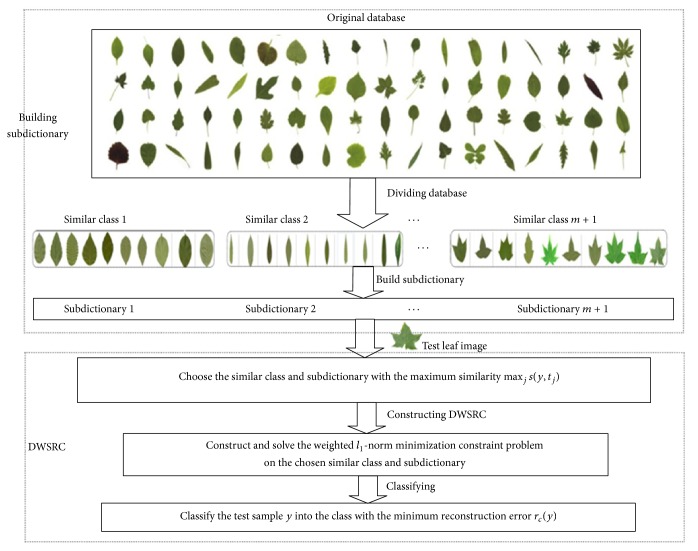
The flowchart of the proposed method.

DWSRC can generate more discriminative sparse codes which can be used to represent the test sample more robustly by combining both linearity and locality information for improving recognition performance. DWSRC is a constrained LASSO problem [21]. Its flowchart is shown in Figure 3.

#### 3.2.1. Feasibility Analysis

In classical SRC, the computational complexity to obtain the SR coefficients is about *O*(*k*^2^*n*) per test sample [14, 15, 17], where *k* is the number of nonzero entries in reconstruction coefficients. Ideally, all of the nonzero SR coefficients will be associated with the columns of the overcomplete dictionary from a single object class, and the test sample can be easily assigned to that class. However, in fact in the training leaf image set, noise, outliers, modeling error, and a lot of intraclass dissimilar leaves, may lead to many small nonzero SR coefficients associated with multiple species classes. The computational complexity to obtain the SR coefficients is nearly *O*(*n*^3^) per test sample [17]. In DWSRC, all of *n* training samples are split into *m* similar classes that are used to build *m* subdictionaries, and a candidate subdictionary is chosen for WSRC. Although the number of the training samples from the candidate similar class is less than the number of all the training samples, the dictionary completeness may not be destroyed, because the candidate training samples that are similar to the test sample are retained, while the noise, outliers, and irregular samples that are dissimilar to the test sample are excluded. The SR coefficients obtained by DWSRC might not be as sparse as the ones achieved by the classical SRC and WSRC, but the SR coefficients of the relevant training samples still own greater magnitudes, because all training samples of a candidate similar class generally play an important role in sparsely representing the test sample.

If we consider all SR coefficients of all similar classes except the candidate similar class to be zeros, we also think DWSRC is conducted on whole the database. Thus, DWSRC is much sparser than SRC and WSRC. On the whole, in DWSRC, Gaussian kernel distance information is imposed on the WSRC to suppress the small nonzero coefficients, so the number of small nonzero SR coefficients of DWSRC should be significantly reduced. The sample number in the candidate similar class is about *n*/*m*. Compared with SRC and WSRC, the computational complexity of DWSRC to obtain the SR coefficients is about *O*(*k*_1_^2^*n*/*m*) per test sample, where *k*_1_ is the number of nonzero entries in reconstruction coefficients by DWSRC. In general, *k*_1_ is much less than *k* and *n*, so the computational complexity of DWSRC is quite less than that of SRC and WSRC.

From the above analysis, it is indicated that DWSR is more suitable for the large-scale database classification problem.

## 4. Experiments and Results

In this section, we investigate the performance of the proposed DWSRC method for plant species recognition and compare it with four state-of-the-art approaches, including plant species recognition using WSRC [21], Leaf Margin Sequences (LMS) [28], Manifold–Manifold Distance (MMD) [29], and Centroid Contour Distance (CCD) [30]. In WSRC based experiments, the parameter selection, data processing and recognition process are similar to that in DWSRC, while in LMS, MMD and CCD based experiments, the parameter selection, data processing, and recognition process are from the three references [28–30], respectively.

The important parameters in DWSRC include the scalar regularization parameter *μ*, two thresholds *T* and *T*_1_, and two Gaussian kernel widths *β* and *β*_1_ in (4) and (6). Among them, as *μ*, *β*, and *β*_1_ are not sensitive to recognition results [13, 21, 31], *μ* in (5) is set to 0.001 empirically, and two Gaussian kernel widths are set as

(9) β=dmaxxi,tj+dminxi,tj2,β1=dmaxy,xri+dminy,xri2,

where *d*_max_(*x*_*i*_, *t*_*j*_) and *d*_min_(*x*_*i*_, *t*_*j*_) are maximum and minimum Euclidean distance between *x*_*i*_ and *t*_*j*_ and *d*_max_(*y*, *x*_*ri*_) and *d*_min_(*y*, *x*_*ri*_) are maximum and minimum Euclidean distance between *y* and *x*_*ri*_.

All experiments are performed on the platform with 3.0 GHz CPU and 4.0 GB RAM by MATLAB 2011b. The MATLAB SR toolbox 1.9 (https://sites.google.com/site/sparsereptool) is used to solve the weighted *l*_1_-norm minimization problem.

### 4.1. Data Preparation

**Figure 4:**
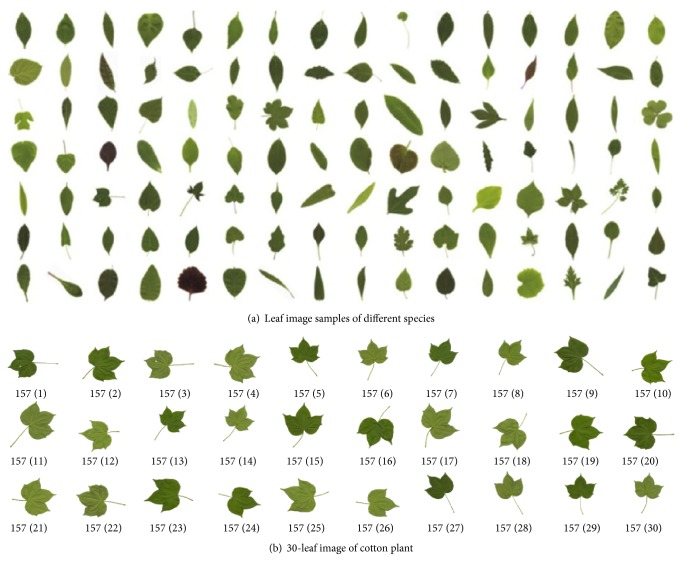
Leaf image samples of ICL database.

All plant species recognition experiments by DWSRC and four competitive methods are conducted on the ICL Leaf database (https://pan.baidu.com/share/link?shareid=2093145911&uk=1395063007), which was collected by intelligent computing laboratory (ICL) of Chinese Academy of Sciences. The database contains 16,846 plant leaf images from 220 kinds of plant species, with different numbers of leaf images per species and different sizes from min-size of 29 × 21 to max-size of 124 × 107. Some examples are shown in Figure 4.

From Figure 4, we find that each leaf image in ICL is single with simple background and without obscureness, twist, and noise, so we ignore a lot of complex preprocessing, such as filtering and enhancement. But, from Figure 4(a), it can be seen that the original leaf images in ICL database are oriented at a random angle and have various shapes, colors, and sizes. Some leaf images contain footstalks, and some kinds of leaves are similar to each other. Because the sizes of the original color leaf images are different from each other, we firstly transform each original image to grayscale image and geometrically scale it to the same size by bilinear interpolation algorithm. From Figure 4(b), it is found that 30 cotton plant leaves have large intraclass difference with low-intensity white backgrounds. To improve recognition rate, several preprocessing steps prior to species recognition are employed as follows:

(1) Convert each color leaf image into grayscale image.

(2) Separate the background from the leaf image by a small threshold (we set as 30); that is, the pixel value less than 30 is set as 0 under gray level of 255.

(3) Reduce noise by a median filter of radius 10.

(4) Cut footstalks off through contour information to detect narrow parallel running chains and remove the longest chain [6, 30].

(5) In order to align all leaf images, extract the angle from the image by which the major axis of the leaf is oriented with respect to the vertical line, and then rotate the image by the angle so that the major axis is aligned with the vertical line; finally remove the white bounding rectangle to superimpose the leaf over a homogeneous background.

(6) Extract leaf contour by Canny Edge algorithm [30].

(7) Crop each contour into the size to contain only the extents of the leaf and then resize them to a square size of 32 × 32 by bilinear interpolation algorithm.

**Figure 5:**
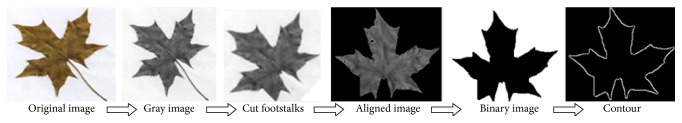
The leaf preprocessing example.

Figure 5 shows a leaf preprocessing example by taking the above steps.

### 4.2. Subdictionary Construction

**Figure 6:**
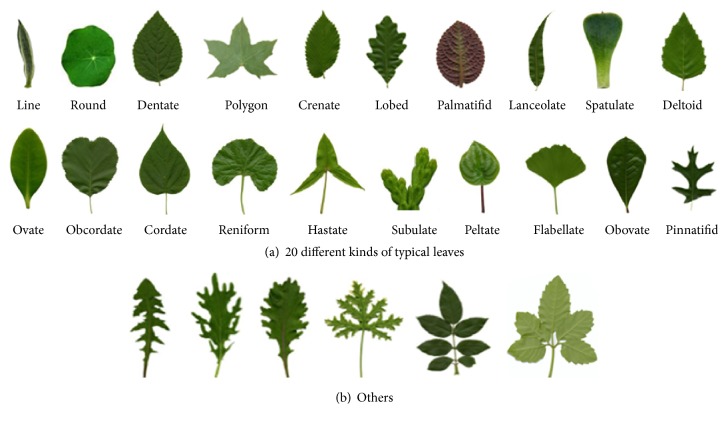
21 kinds of typical leaves.

From Figure 4(a), we see there are innumerable various plant leaves, but these leaves can be clustered into several similar classes by leaf shape description or similarity measure, such as Acerose, Linear, Gladiate, Ensiform, Oblanceolate, Ovate, Oval, Obovate, and Elliptic. The number of the classes can be defined by* K*-mean clustering and* K*-fusion clustering algorithms [32]. We referred to two websites, http://theseedsite.co.uk/leafshapes.html and https://en.wikipedia.org/wiki/Glossary_of_leaf_morphology, and four references [33–36] and defined the class number as 21. In fact in nature, we can choose 20 kinds of typical leaves, as shown in Figure 6, and find the similar leaves of each typical leaf from the ICL database. Then we obtain 21 similar classes.

**Figure 7:**
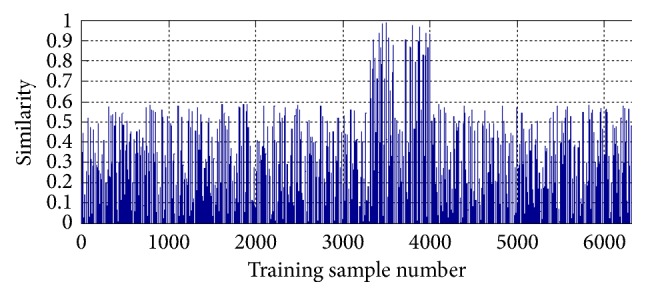
Partial similarities between the dentate leaf image and each image of the ICL database.

Suppose *t*_*i*_  (*i* = 1,2,…, 20) is the *i*th representative leaf image, *x*_*j*_  (*j* = 1,2,…, 16846) is the* j*th leaf image of the ICL database, and the similarity *s*(*t*_*i*_, *x*_*j*_) between *t*_*i*_ and *x*_*j*_ is calculated by (4).  Figure 7 is the similarities between a given dentate shape leaf image and each leaf image of the ICL database. In order to clearly show the visual graph of selecting the candidate training leaf images, Figure 7 only shows partial results, that is, about 6300 similarities.

From Figure 7, we can observe that a few leaf images are similar to the given test leaf image. We set the threshold *T* = 0.5; that is, if *s*(*t*_*i*_, *x*_*j*_) ≥ 0.5, classify *x*_*j*_ into the *i*th similar class. After most of leaf images of the ICL database are divided into 20 similar classes, the remaining images are clustered into the 21st similar class. Finally, we build 21 subdictionaries by stacking all preprocessed contours of each similar class as columns of a matrix, respectively.

### 4.3. Classifying Plant Species by DWSRC

Given a new test leaf image *y*, we calculate the similarity between *y* and each typical leaf *t*_*i*_  (*i* = 1,2,…, 20). We set the second threshold *T*_1_ = 0.1, that is, if *s*(*y*, *t*_*j*_) < 0.1 for any *j* = 1,2,…, 20, and select the 21st similar class as the candidate similar class and *A*_21_ as candidate subdictionary. Otherwise, we select the candidate similar class and candidate subdictionary with the maximum similarity *s*(*y*, *t*_*j*_)  (*j* = 1,2,…, 20).

**Figure 8:**
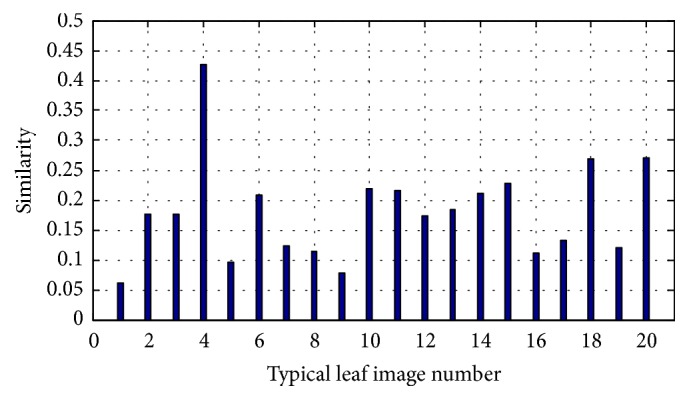
The similarity between *y* and each typical leaf *t*_*i*_  (*i* = 1,2,…, 20), where *y* is cotton leaf.

Figure 8 is a similarity example, where the test image is a cotton plant leaf image. From Figure 8, the maximum similarity is *s*(*t*_4_, *y*) = 0.4269. Then we choose the fourth similar class and the fourth subdictionary *A*_4_ to construct DWSRC for classifying the test image. We calculate the SR coefficients and recognize the test leaf by the minimum reconstruction error.

Figures 7 and 8 only list the visual process on how to divide the whole dataset into several similar classes and construct the subdictionary for the SRC algorithm, where the training set is all images of ICL database, and the test set is any leaf image.

### 4.4. Experimental Results

To illustrate how DWSRC and 4 competitive methods work, two kinds of experiments are conducted:

(1) From ICL database, we randomly select 1,100 images from 220 species with 5 images per plant species as the test set and the rest as the training set. The training set is divided into 21 similar classes to build the subdictionaries, and each test leaf is recognized by DWSRC, where the typical samples are shown in Figure 6(a). The training and test random partition is repeated 50 times. The average recognition results of 50 experiments of five methods are shown in Table 1.

(2) From ICL database, we randomly select 2,000 images from 16,846 plant leaf images as the test set and the rest as the training set and repeat the training and testing procedure 50 times. In each time, the training set is also divided into 21 similar classes to build the subdictionaries, and each test leaf is recognized by DWSRC, where the typical samples are also shown in Figure 6(a). The average recognition results of 50 experiments of five methods are shown in Table 2.

In Tables 1 and 2, the bold fonts highlight the best recognition performance. From two tables, we can see that the recognition performance of DWSRC is the best. The main reason might be that DWSRC employs only the candidate similar class to solve the SR problem and recognize the test samples, and it makes use of the similarity relationship between the test sample and each training sample. So the computational cost is significantly reduced and the side-effect of outliers, noise, and modeling error to the recognition performance is greatly suppressed. The recognition rate of WSRC is higher than LMS, MMD, and CCD, but its computational time is the longest. Most of the time is taken to solve the *l*_1_-norm minimization problem. Since LMS, MMD, and CCD need to extract the classifying features from each leaf image, which is time-consuming, they have lower recognition rates than WSRC and DWSRC. Because the real-world leaves of the same species are different from each other, it is difficult to extract the “optimal” features from each leaf image.

## 5. Conclusions

As for the large-scale plant species recognition issue, we proposed a new SRC approach, that is, discriminant weighted SRC (DWSRC), which aims to exploit the locality and similarity of the original dataset and the test samples in sparsely representing the test samples. It makes use of the prior structure information of the dataset and the distance between the test sample and training samples to construct the SR problem. To represent a test sample, we not only consider the original data structure of training samples, but also take into account the locality and the similarity between the test sample and each training sample. The SR coefficients of the test sample maintain a sparse structure and more discriminative information. DWSRC is essentially the generalization of WSRC. Rather than WSRC, we construct several subdictionaries prior to SRC by dividing all training samples into several similar classes and then choose a candidate subdictionary for given test sample to implement WSRC. Thus, the computational cost to solve the *l*_1_-norm minimization problem is greatly reduced. In this sense, DWSRC is reasonable for large-scale plant species recognition, which can improve the plant species recognition rate and reduce the computation cost. Recently, deep learning has led to a series of breakthroughs in many pattern recognition research areas. We will study deep learning for leaf based species recognition in the future work.

## Figures and Tables

**Table 1:** Average recognition rates, standard deviation, and running time of WSRC, LMS, MMD, CCD, and DWSRC.

Method	WSRC	LMS	MMD	CCD	DWSRC
Recognition results	88.27 ± 1.89	82.73 ± 1.73	86.51 ± 1.46	81.62 ± 2.53	**91.12 ** **± 1.25**
Running time (s)	1218	842	936	723	**437**

**Table 2:** Average recognition rates, standard deviation, and running time of WSRC, LMS, MMD, CCD, and DWSRC.

Method	WSRC	LMS	MMD	CCD	DWSRC
Recognition results	86.56 ± 2.12	82.50 ± 2.06	85.43 ± 1.52	81.17 ± 2.56	**90.64 ** **± 1.42**
Running time (s)	1432	953	1016	647	**516**
